# Five Dimensions of European Identity: A Contribution to the Italian Adaptation and Validation of the In-Group Identification Scale

**DOI:** 10.5964/ejop.v12i2.1058

**Published:** 2016-05-31

**Authors:** Francesco La Barbera, Vincenza Capone

**Affiliations:** a Department of Political Science, University of Napoli “Federico II”, Naples, Italy; b Department of Humanities, University of Napoli “Federico” II, Naples, Italy; Aristotle University of Thessaloniki, Thessaloniki, Greece

**Keywords:** in-group identification, multi-dimensional identity, European identity, Italian adaptation, SEM

## Abstract

Recent approaches define collective identity as a multi-component construct. Nonetheless, there is a lack of research about the dimensionality of in-group identification in relation to European Identity. Leach and colleagues (2008) proposed a framework of in-group identification, in which they distinguish five main components integrated into two higher-order dimensions. In two studies we examined the validity of the Italian version of the In-Group Identification Scale by Leach et al., with a focus on European identity. Confirmatory factor analyses revealed that the hierarchical model of in-group identification fitted the data well (Study 1); the measure was shown to have satisfactory convergent and divergent validity. In Study 2, the relations between European identification and several possible antecedents and outcomes were examined.

There are several major theories in social sciences which address the topic of the individuals’ identification with a collective entity, such as a group or a social category; collective identity has been shown to have huge implications for people attitudes and behaviour (Ashmore, Deaux, & McLaughlin-Volpe, 2004; Cartwright & Zander, 1968; Tajfel & Turner, 1979).

The degree to which an individual identifies with an in-group determines the degree to which that membership is psychologically and socially influent (Ellemers, Spears, & Doosje, 1999). Therefore, many scholars, in the last decades, have devoted their work to the conceptual definition and measurement of in-group identification (see Ashmore et al., 2004; Capozza & Brown, 2000).

In social psychology, Tajfel’s influential approach defines social identity as “that part of an individual’s self-concept which derives from his knowledge of his membership of a social group together with the value and emotional significance attached to that membership” (Tajfel, 1978, p. 63). Hence, social identity has been originally proposed as a multi-dimensional construct, with a cognitive, an evaluative, and an affective dimension (Tajfel, 1978, 1981). Nonetheless, identification has been often intended as a general connection to an in-group and measured by a mono-dimensional scale, yet this approach has been criticized (Leach et al., 2008).

Consistent with Tajfel’s original definition, recent approaches propose social identity as a multidimensional construct, and a number of conceptualizations and measures have been elaborated (Cameron, 2004; Jackson, 2002; Leach et al., 2008; Luhtanen & Crocker, 1992; Obst & White, 2005). Research has shown that individuals identify themselves to a different degree in relation to different identity dimensions; in addition, social identity dimensions have been found to be differently predictive of attitudes and behaviour (Ellemers, Kortekaas, & Ouwerkerk, 1999; La Barbera & Cariota Ferrara, 2012). However, as underlined by Ashmore and colleagues (2004), the number and characteristics of the in-group identification dimensions are still unclear; the proliferation of theories and measures has determined a somewhat confusing scenario, in which “the same label can be used to refer to different concepts, or similar concepts are used with different labels” (p. 82).

To address this issue, Leach and colleagues (2008), on the basis of a wide review of the existing conceptualizations and measures of in-group identification, have proposed a new theoretical framework, in which they distinguish five main components integrated into two more abstract, higher-order dimensions: group-level self-definition and group-level self-investment. The five components are: 1) individual self-stereotyping, 2) in-group homogeneity, 3) solidarity, 4) satisfaction, and 5) centrality.

As Lovakov, Agadullina, and Osin (2015, p. 3) state: “Individual self-stereotyping is the degree to which an individual perceives herself as similar to an in-group prototype. In-group homogeneity is the degree to which an individual perceives her in-group as relatively homogeneous and distinct from relevant out-groups”. Individual self-stereotyping and in-group homogeneity are, in turn, two dimensions of the group-level self-definition factor. The group-level self-investment factor organizes the remaining three components. Solidarity refers to “a sense of belonging, a psychological attachment to the in-group, and coordination with other group members. Satisfaction refers to the positive evaluation of the in-group. Centrality is the salience and importance of in-group membership” (Lovakov et al., 2015, p. 3).

Leach and colleagues (2008) operationalized the hierarchical model proposed by a 14 item scale—the In-Group Identification Scale. This measure gives a complete view of group identity, and has robust psychometric properties. Recently, Roth and Mazziotta (2015) adapted and validated the scales on a German large sample, in relation to three different identification targets (gender, nation, organization). Results confirmed the good psychometrics quality of the instrument.

The current study primarily aims to evaluate the validity of the Italian version of the In-Group Identification Scale, examining the structure, reliability, convergent, divergent, and construct validity. In addition, we choose to conduct our analyses with a focus on a more challenging identity, namely the European identity. In two studies, indeed, participants were asked about their identification as Europeans. There is a huge interest among social scientist about European identity (Castano, 2004; Herrmann & Brewer, 2004; Hooghe & Marks, 2004; Lubbers, 2008; Thomassen & Back, 2008; Vetik, Nimmerfelft, & Taru, 2006), which has been shown to have a significant effect on relevant outcomes, such as group-based trust, willingness to cooperate, attitudes towards EU deepening and widening (La Barbera, 2015; La Barbera, Cariota Ferrara, & Boza, 2014; Zielonka, 2005). Moreover, there is a growing interest about the dimensionality of European identity, and few theoretical conceptualization has been proposed (Bruter, 2003; La Barbera & Cariota Ferrara, 2012), yet the topic needs a deeper investigation (La Barbera et al., 2014). Leach and colleagues’ (2008) approach could be particularly interesting and useful in this context, to open up a research line aimed to clarify the number, quality, antecedents and effects of the different dimensions of European identity. Therefore, our study aimed to give a first contribution in this direction, examining the psychometric properties of the Italian version of in-group identification measure by Leach and colleagues (2008) in relation to European identity (Study 1), and exploring how the different dimensions of European identity are correlated with established antecedent and dependent variables (Study 2).

## Study 1

Leach and colleagues (2008) identified five different components of in-group identification integrated into two more abstract, higher-order dimensions: we expected to confirm the same hierarchical structure in the Italian sample. We also hypothesized the five subscales of the Italian In-Group Identification Scale to have a high internal reliability, similar to earlier findings in the Leach and colleagues’ samples.

We hypothesized that our studies would have confirmed the convergent and divergent validity of the Italian In-Group Identification Scale, with its subscales correlating with corresponding measures. Thus we expected all five sub-dimensions of the scale to be correlated with a general measure of in-group identification (Doosje, Ellemers, & Spears, 1995). On the other hand, for testing divergent validity, we needed a construct that could be supposed to be in contrast with all the five identity dimensions. Previous research has steadily shown a positive relationship between ingroup identification and positive affect towards the ingroup (e.g. Brewer, 1999; Cameron, 2004). Therefore, we hypothesized the five components of in-group identification to correlate negatively with a measure of in-group negative affect we build *ad hoc* for the EU context.

### Method

#### Participants

Privacy information and consent to the processing of personal data in accordance with the international ethical standards were asked to participants. As a guarantee of anonymity, each questionnaire was marked by an alphanumeric identification code, previously assigned to each participant.

A total of 210 pencil-and-paper questionnaires were distributed, and 195 were returned (response rate: 92.9%). The participants were Italian University Students in Political Science, between the ages of 18 and 48 (*M* = 21.52 years old; *SD* = 4.46), with 62.8% identifying as female. All participants indicated that they were born in Italy and that their native language was Italian. The data were provided by students in Italy within the context of their university. Items were presented in a single random order.

#### Measures

##### The Italian In-Group Identification Scale

The Group Identification Scale by Leach and colleagues (2008) was used; the scale consists of 14 items organized into five sub-scales: Individual Self-Stereotyping (ISS, 2 items), In-Group Homogeneity (IGH, 2 items), Satisfaction (4 items), Solidarity (3 items) and Centrality (3 items). Example of items are: ‘I feel a bond with Europeans’; ‘I am glad to be European’.

The Italian translated In-Group Identification Scale was back-translated to ensure translation equivalency. Two bilingual PhD researchers were involved in the translation process. One of the researchers who translated the original tool to Italian had a PhD in Social Psychology from a university in Italy. The other translator, who had been educated in the United States and had a PhD in Psychology, translated the Italian In-Group Identification Scale back to English, without any discussion with the first translator. Adjustments were provided for ensuring understandability, psychological equivalence, and the correctness of the translation from English to Italian. The original and back-translated English versions did not differ appreciably as judged by the translators. Participants were asked to indicate their agreement on a scale from 1 (totally disagree), to 10 (totally agree).

##### Group Identification Scale

The Group Identification Scale by Doosje and colleagues (1995) was used. This measure consists of 4 items (e.g. ‘‘I see myself as an European’’). Participants were asked to rate each item on a scale from 1 (totally disagree), to 10 (totally agree). The whole scale had a reliability of .71.

##### Negative Affect Scale

Drawing on previous studies (Boomgaarden, Schuck, Elenbaas, & de Vreese, 2011; La Barbera & Cariota Ferrara, 2012), five items were used for measuring participants’ negative feelings about EU (e.g.: “I feel threatened by European Union”). Participants indicated their agreement with each item on a scale from 1 (totally disagree), to 10 (totally agree). The internal coherence proved adequate, α = 0.82.

### Results and Discussion

In the first study, the items of In-Group Identification Scale were evaluated with regards to variance and frequency distribution, in order to select the items for the factor analysis. The Kaiser–Meyer–Olkin (KMO) measure of sampling adequacy and Bartlett’s test of Sphericity were considered to test whether the dataset was suitable for factor analysis. Confirmatory factor analysis (CFA) was conducted, using the maximum likelihood estimation method, to evaluate the underlying structure of items. In order to evaluate the solution we took into account the goodness of fit indexes. The Chi-square (χ*^2^*) was used to indicate the difference between observed and expected covariance matrices, testing the null hypothesis of ideal model fit where the residual covariance equals zero. We also considered adequate: Comparative fit index (CFI) and the Tucker–Lewis index (TLI) values above 0.90 (Bentler, 2005; Byrne, 1994), Root mean square error of approximation (RMSEA) values below or equal to 0.06, and Root mean square residual (RMSR), values equal to or below 0.09 (Hu & Bentler, 1999). To compare models on the basis of the same data matrix compare models fit, we used the Akaike information criterion (AIC) index (Kline, 2000; Byrne, 2011).

In order to test the reliability of the scale, we computed the internal consistency using Cronbach’s alpha. An internal consistency greater than 0.70 is thought to be necessary for a valid psychological scale (Nunnally & Bernstein, 1995). For the analysis of the internal consistency of the scale, the corrected correlation between the score of the item and the second order dimensions of In-Group Identification Scale was computed.

Relations between the measures were examined using the Pearson product–moment correlations. Statistical significance was set at p-value < 0.05. Analyses were computed with SPSS 15.0 and MPLUS 5.0 for CFA.

In order to analyze internal consistency of the Italian In-Group Identification dimensions, we calculated the corrected correlation between the score of the items and each of second order dimensions. Maximum likelihood estimation was used in all the analyses. For “Self-definition” coefficients ranged between 0.51 (Item 1) and 0.73 (Item 4) and were considered adequate since they were greater than 0.30 (Nunnally & Bernstein 1995). The means of the items ranged from 4.97 (Item 4) to 5.57 (Item 1). The standard deviation was higher than 1 for all items, ranging between 1.92 (Item 2) and 2.34 (Item 3). Skewness and kurtosis showed a normal distribution in items’ response: skewness ranging between -0.32 (Item 1) and 0.30 (Item 4), and kurtosis ranging between -0.63 (Item 2) and 0.23 (Item 1). For “Self-investment” coefficients ranged between 0.57 (Item 10) and 0.82 (Item 14). The means of the items ranged from 3.41 (Item 6) to 6.66 (Item 13). The standard deviation was higher than 1 for all items, ranging between 2.06 (Item 6) and 2.63 (Item 13). Skewness ranging between 0.18 (Item 5) and -0.62 (Item 13), and kurtosis ranging between -0.97 (Item 10) and -0.78 (Item 5).

**Figure 1:**
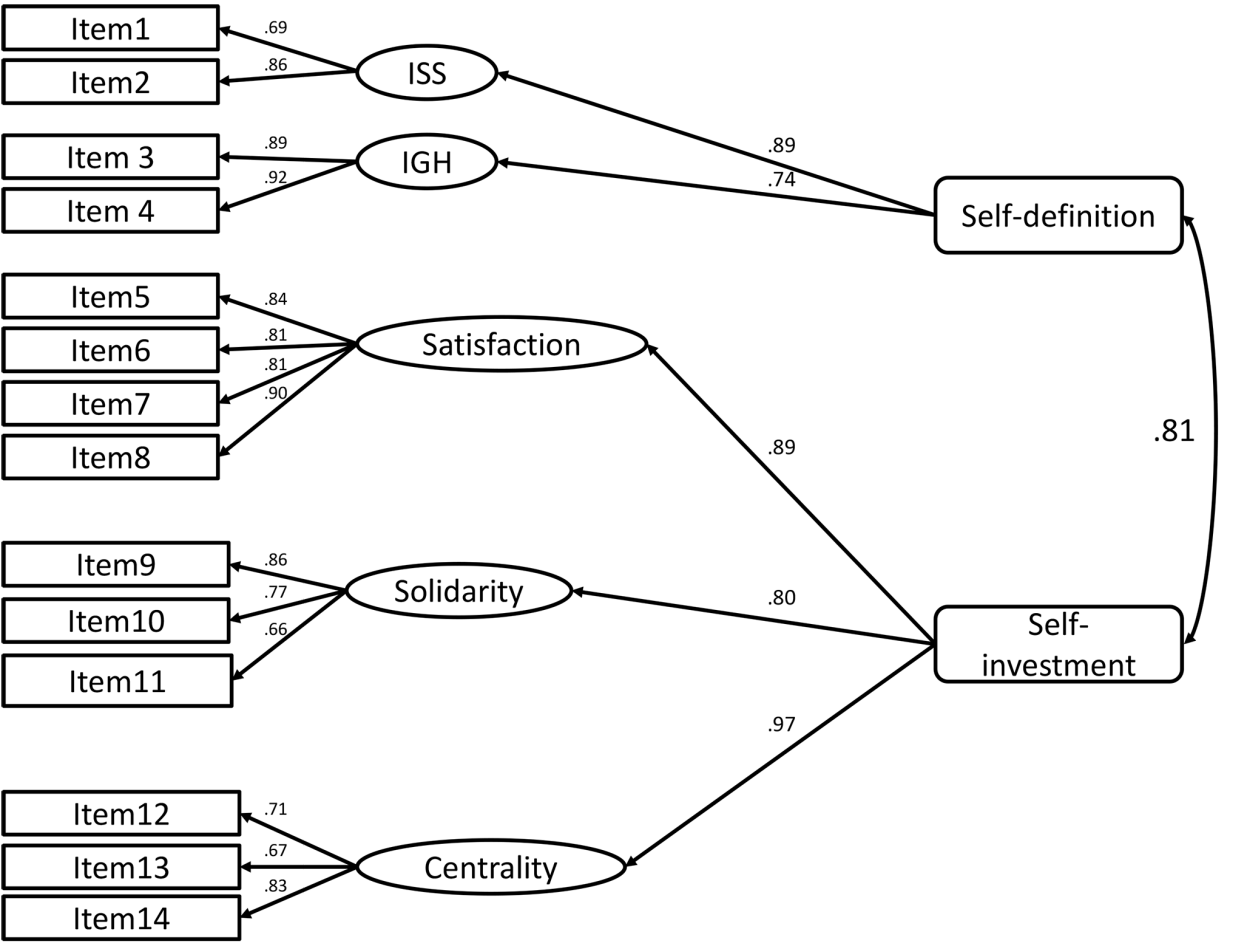
Proposed measurement model for European identification. *Note.* ISS = individual self-stereotyping; IGH = in-group homogeneity. For all coefficients *p* <.001.

To test how well the measurement model fit the 14 items of in-group identification we performed CFAs, following the procedure used by Leach et al. (2008, p. 149) “to examine how well the proposed measurement model fit the 14 items of in-group identification. Items were permitted to load only on the component they were expected to indicate, and no item errors were allowed to correlate. The five components of in-group identification were specified as indicating second-order factors of Self-definition (individual self-stereotyping and in-group homogeneity) or Self-investment (satisfaction, solidarity, centrality). Although the five components were not allowed to correlate, the second-order factors of self-definition and self-investment were allowed to correlate” (see Figure 1). Table 1 shows the fit of this proposed measurement model: it fit the data well for identification as European.

**Table 1:** Fit of Competing (Measurement) Models of In-Group Identification in Study 1

Model	χ^2^(*df*); *p*	RMSEA	90% CI	RMSEA *p* ≤ .05	SRMR	CFI	TLI	AIC
Five-component/two-dimensional (Figure 1)	119.029(71); *p* = .0003	.059	.040, .077	.206	.037	.97	.96	10743.572
Alternative models								
Model a. Five-component/one-dimensional	135.102(72); *p* = .00001	.067	.049, .084	.056	.043	.96	.95	10757.645
Model b. Two-component: Self-definition and self-investment	303.707(76); *p* = .00001	.124	.110, .139	.000	.085	.86	.83	10918.250
Model c. One-component: Identification	440.230(77); *p* = .00001	.156	.142, .170	.000	.079	.78	.74	11052.773
Model d. Two-component: Cognitive/self-categorization and affective ties/social identity	546.081(77); *p* = .00001	.177	.163, .191	.000	.291	.72	.66	11158.625
Model e. Alternative five-component/two-dimensional	135.101(71); *p* = .00001	.068	.050, .085	.047	.043	.96	.95	10759.645

As shown in Figure 1, all standardized item loadings exceeded .45, with many above 0.80, and differed significantly from zero (*p* < .001). Each of the five components loaded onto the expected second-order factor. These loadings exceeded 0.60, and differed significantly from zero (*p* < .001). The second-order factors of self-definition and self-investment were strongly and significantly related (.81 *p* < .001). All these parameters confirm that the model, with five components and two second-order factors, fitted well.

In a second step, we estimated the fit of the five alternative models suggested by Leach et al. (2008). The first alternative model (model a, see Table 1) specified the five components as indicating one second-order factor of in-group identification. This model provided only marginal fit to the data. In addition, it produced AIC values higher than the proposed model.

A second alternative model (model b, see Table 1) posited that: 1) the items of individual self-stereotyping and in-group homogeneity saturated on self-definition factor; 2) the items of centrality, satisfaction, solidarity saturated on self-investment factor. This model fit the data poorly.

A third alternative (model c, see Table 1) specified all of the items as indicating a general factor of in-group identification. As well, this model fit the data very poorly.

A fourth alternative model (model d, see Table 1) posited that: 1) the items of individual self-stereotyping, in-group homogeneity, and centrality saturated on a cognitive/self-categorization factor (see Leach et al., 2008; Jackson, 2002); 2) the items of satisfaction and solidarity saturated on an affective ties/social identity factor. This model fit the data poorly.

The last alternative model (model e, see Table 1) specified the component of individual self-stereotyping, in-group homogeneity, and centrality as saturating on the self-definition dimension, whereas the items of satisfaction and solidarity as saturating on the self-investment dimension. Although this model tended to fit the data satisfactorily, in line with Leach et al.’s (2008) study, its AIC values tended to be higher those of the proposed model, such as RMSEA. Furthermore the Probability RMSEA is significant.

#### Reliability and Descriptive Statistics

Given the confirmation of the five-component measurement model, we computed scale scores for each component. All five scales were of moderate or high reliability, Cronbach’s α were: ISS, *α* = .72; IGH, *α* = .90; Satisfaction, *α* = .81, Centrality, *α* = .79, Solidarity, *α* = .79. Table 2 summarizes the descriptive and correlational statistics obtained on the in-group identification subscales.

**Table 2:** Study 1: Descriptive and Correlational Statistics

Scale	*M*	*SD*	1	2	3	4	5
1. ISS	5.36	1.76	–				
2. IGH	4.84	2.23	.53**	–			
3. Satisfaction	6.09	2.15	.53**	.48**	–		
4. Centrality	4.20	4.20	.50**	.51**	.69**	–	
5. Solidarity	5.62	2.11	.47**	.41**	.61**	.64**	–

**p < .001.

#### Convergent and Divergent Validity

Tables 3 and 4 illustrate the correlations between the five components of in-group identification and the established measures.

**Table 3:** Convergent Validity: Bivariate Correlations With Group Identity Scale (Study 1)

Scale	IGS	IGS (p*r*)
ISS	.63**	.40**
IGH	.55**	.32**
Satisfaction	.74**	-
Cetrality	.76**	.51**
Solidarity	.74**	.54**

*Note.* Satisfaction controlled in partial *r* (p*r*).

**p < .001.

**Table 4:** Divergent Validity: Bivariate Correlations With Group Identity Scale (Study 1)

Scale	Negative affect	Negative affect (p*r*)
ISS	-.47**	-.26**
IGH	-.28**	-.04
Satisfaction	-.53**	-
Cetrality	-.32**	-.07
Solidarity	-.40**	-.12

*Note.* Satisfaction controlled in partial r (p*r*).

**p < .001.

For the sake of comparability with Leach et al. (2008), we calculated also the partial correlations which control for satisfaction, because satisfaction is a general and strong component of in-group identification and tends to correlate most highly with the different scales.

As we expected, all five dimensions of In-Group Identification Scale were significantly interrelated with IGS, either controlling or not for satisfaction. This supported the convergent validity of the scale tested. On the other hand, consistent with our hypothesis, the correlations between the five components of in-group identification and negative affect were significant and negative; controlling for satisfaction, correlations remained negative, yet dropped in significance, except for individual self-stereotyping, whose negative correlation with IGIS was significant and low. Overall, results suggested divergent validity of the five scales.

## Study 2

In second study, our aim was to expand on the Leach et al. (2008) studies exploring the relation between in-group identification and some of its possible antecedents and outcomes. Indeed, the (empirical) validity of distinguishing between different dimensions of a constructs is corroborated by finding that relevant variables are differently interrelated with them (see La Barbera & Cariota Ferrara, 2010). In addition, in second study we had a keen interest to several variables relevant to the EU context. In recent years, several studies have addressed the relationships between people’s identification as Europeans and psycho-social factors such as cooperation and pro-European attitudes (La Barbera, 2015; La Barbera, Cariota Ferrara, & Boza, 2014). Nonetheless, the identification as Europeans has almost been measured by uni-dimensional scales (for an exception, see La Barbera & Cariota Ferrara, 2012). Therefore, our second study provides an early exploration of the complex issue of relationships between different dimensions of European identity and their possible antecedents and consequences.

We expected the gender of participants to affect the solidarity dimension of in-group identification, considering that gender research suggests that women excel in the relational domain (Brands & Kilduff, 2014), in part because social activity matches prevailing stereotypes concerning women as communal and warm human beings (Spence & Buckner 2000). Women tend to rate themselves more highly than men on self-related dimensions concerning interdependence; in terms of conversational norms, they prefer talking about relationships when describe themselves (Cross & Madson, 1997). For women, the significance of the group results from the degree to which the group fulfills their relational needs (Maddux & Brewer, 2005).

On the contrary, we expect perceived interdependence to affect all five dimensions. Indeed, previous research has shown that interdependence feeling affects either group level self-definition and group-level self-investment (Henry, Arrow, & Carini, 1999; Leach et al., 2008; Lewin, 1948; see also Capone, Petrillo, & Romano, 2013).

As dependent variables potentially affected by participants’ identification with EU, we focused on two dependent variables which have been the target of an emergent research (Boomgaarden, Schuck, Elenbaas, & de Vreese, 2011; La Barbera, 2015; Schuck & de Vreese, 2006): the attitudes towards EU integration and EU enlargement. EU integration (“deepening”) and enlargement (“widening”) have been considered as two different variables (Karp & Bowler, 2006), taking into account that in countries such as the UK, EU enlargement is supported, whereas EU integration is not (Lubbers & Scheepers, 2010).

The attitude towards EU integration is the orientation towards the increase of EU power, even in substitution of national sovereignty, and it is related to EU legitimacy (Castano, 2004). The EU widening addresses the issue of demarcation: who is a group member, and who is not (Maier & Rittberger, 2008)?

However, either the attitude towards EU deepening and the attitude towards EU widening have been found to be positively correlated with identification with EU (Boomgaarden et al., 2011; de Vreese & Boomgaarden, 2006; Fuchs & Schlenker, 2006; La Barbera, 2015). Nonetheless, as we already stated, there is a lack of research about the different dimensions of European identity and, in our best knowledge, any research has addressed the issue of which dimension of identification with EU would affect those attitudes. Therefore, in second study we provided an early exploration of that issue.

Taking into account that the EU deepening recalls the comparison between memberships at different levels (local, national, supra-national), we hypothesized that centrality would be correlated with that attitude more than the other dimensions.

As for EU widening, we expected instead the satisfaction to play a significant role. The less individuals are satisfied with their in-group experience and status, the less likely they are willing to welcome new members (Tajfel & Turner, 1979), who could represent a threat for the group.

### Method

#### Participants

The second study sample consisted of 89 undergraduate college students (50 women) enrolled in a course of Agricultural at the University “Federico II” of Naples. The mean age for this group was 24.41 years, *SD* = 3.77, range from 20 to 40.

As in Study 1, research participation complies with the international ethical standards. The data were provided by students in Italy within the context of their university. Items were presented in a single random order.

#### Measures

##### Interdependence

Participants were asked to indicate their agreement (on a scale from 1 to 10) with the following statement: “The member States of European Union share a common fate”.

##### Attitude towards EU deepening

Five items (see Appendix 1) were used for measuring participants’ attitude towards EU deepening (La Barbera, 2015). Participants indicated their agreement on scales from 1 (totally disagree) to 10 (totally agree). The measure had adequate internal coherence (*α* = .72), and a mean score for the whole scale was computed, with higher values corresponding to a more favourable attitude towards EU deepening.

##### Attitude towards EU widening

Three items (see appendix 1) were used for measuring participants’ attitude towards EU enlargement (La Barbera, 2015). Participants indicated their agreement on scales from 1 (totally disagree) to 10 (totally agree). The whole scale had good internal coherence (*α* = .81), and an average score was computed, with higher values corresponding to a more favourable attitude towards EU enlargement.

### Results and Discussion

Table 5 provide males’ and females’ mean scores on the identification scales. As we hypothesized, the effect of gender was significant only for the solidarity component of identification, *t*(87) = 2.05, *p* < .05 (for the other components, *t*s < 1, *p*s > .10).

**Table 5:** Study 2: Identification Scores by Gender and Interdependence Level

Scale	Gender		Interdependence level	
	Male	Female	Low	High
ISS	3.93	4.25	3.72	4.52
IGH	3.87	3.95	3.32	4.48
Satisfaction	4.58	4.73	4.24	5.10
Cetrality	3.22	3.10	2.67	3.61
Solidarity	3.89	4.46	3.69	4.79

Using a median split procedure, we divided participants in two groups with relatively high vs. low scores on the interdependence item. Then we compared the mean scores of the two groups on the five identification scales (see Table 5). As we expected, the identification mean scores of participants in the high interdependence group, compared to those in the low interdependence group, were higher on all five dimensions, *t*s > 3, *p*s < .01.

Finally, Table 6 shows the interrelations between the five identification scales and the attitudes towards EU deepening and widening.

**Table 6:** Intercorrelations Between Identification and Attitudes Towards EU Deepening and Widening

Scale	Deepening	Widening
ISS	.135	.225*
IGH	.170	.319**
Satisfaction	.171	.369**
Centrality	.247*	.326**
Solidarity	.084	.203

*p < .05. **p < .01.

As for EU deepening, we found a significant correlation only with the centrality subscale, which support our hypothesis. Further, as we expected, satisfaction was significantly correlated with attitude towards EU widening; EU widening was also significantly correlated with centrality, self-stereotyping and in-group homogeneity.

## General Discussion

The central aim of this study was to examine the psychometric properties of the Italian version of in-group identification measure by Leach et al. (2008). In meeting this goal, we conducted two studies. In Study 1, the CFA shown that the hierarchical model of in-group identification, which included the second-order factors of group-level self-definition (individual self-stereotyping and in-group homogeneity) and group-level self-investment (satisfaction, centrality and solidarity) fitted the data well and showed a better fit than the alternative models. We examined the construct validity and reliability of the Italian version of in-group identification measure: findings highlight that these scales have good psychometric properties. All items showed their highest factor loadings with the dimensions, suggesting that the 14 items of the Italian version of the Leach and colleagues’ measure of in-group identification are adequate. The measure was shown to have satisfactory convergent and divergent validity.

In addition, the current paper contributes to extend the Leach et al. (2008) study in several ways, apart from being the first study of an Italian sample.

First, we used different validation measures than the previous study, especially including measures for negative affect to test the divergent validity. Second, the Likert-type response scale that ranged from 1 (strongly disagree) to 7 (strongly agree) used in the original study was considered useful, yet not much discriminating. Therefore, we preferred a rating scale with 10 response categories; the psychometrics properties of the original scale do not seem weakened. The results of our first study provide solid support for Leach et al. (2008) concept of in-group identification as a multidimensional construct: the hierarchical model of in-group identification can be a useful tool in future research.

In Study 2, we explored the interrelations of the five identification scales with several antecedent and outcome variables. Although limited by the small sample, findings offer some interesting points. The five scales appear to be differently interrelated with several (independent and dependent) variables, namely the participants’ gender and their attitudes towards EU deepening and widening. These results support Leach et al. (2008) scale’s construct validity, also providing further insight into the advantages of using a multidimensional measure of in-group identification rather than a uni-dimensional measure. Recent studies have proposed measures of identification which consist of a single item (Postmes, Haslam, & Jans, 2013; Reysen et al., 2013). As the authors state, these simple measures could be very useful, especially in research contexts with limited resources in terms of time and data-processing costs; nonetheless, “multidimensional measures are recommended for researchers interested in the associations between a phenomenon and specific dimensions of in-group identification” (Reysen et al., 2013, p. 469). However, the idea of measuring identification with a single item relies on the proposed homogeneity of the psychological construct, which should be well represented by a uni-dimensional model. This is not supported by our findings, showing that Leach et al. 2008’s hierarchical model fit the data better than a uni-dimensional model (see also Leach et al., 2008; Roth & Mazziotta, 2015).

Although results for the Italian version of the measure by Leach and colleagues (2008) are promising, there are some limitations to the present research. The Italian version measure of in-group identification has been investigated only in one type of in-group. Moreover, identification with Europe refers to a social group with a low degree of interaction between group members. Hence, the model of in-group identification needs to be validated with different groups (e.g. task-oriented, primary groups). The concurrent and divergent validation of in-group identification is not complete: there are a number of different constructs that could have been measured in order to test the validity of the measure. The sample of Study 2 is quite small, although similar sized sample have been previously used for testing scales’ predictive validity (e.g., Reysen et al., 2013, Study 3). Increasing the sample size and heterogeneity could give more opportunity to detect significant relationships between the identification dimensions, their antecedents, and the relevant psycho-social variables they affect. Finally, a longitudinal design could be employed in future research to investigate test-retest reliability, which has not been explored either in the original or in the adapted version.

As regards the EU context, present research provide a first contribution to the exploration of the dimensionality of European Identity, a crucial topic on which there is a growing interest (e.g., Bruter, 2003; La Barbera & Cariota Ferrara, 2012), yet there is not a significant empirical evidence on the number and quality of European identity dimensions. Leach et al. (2008)’s studies could provide a framework to better understand the structure of citizens’ identification with EU. For example, as we hypothesized, our results show that only the centrality dimension is correlated to attitude towards EU deepening. Indeed, EU further integration involves comparisons and sometimes contrasts between peoples’ memberships at different levels (local, national, supra-national). Therefore, individuals’ identification with EU plays a significant role in fostering their attitude towards EU deepening when the European membership plays a significant role in their identity hierarchy. In a similar vein, using Leach and colleagues’ theoretical framework and instruments, future research could fruitfully investigate the interrelation between the different components of identification with EU and other variables relevant to the EU context.

## Appendix: Scales Used in the Study

**Strength of identification with EU (adapted from Spears, Doosje, & Ellemers, 1997)**
I think about myself as a European citizen.
I feel I am deeply tied to other European citizens.
On the whole, I am glad to be a European citizen.
Being a European citizen fully represents me.

**Attitude towards EU integration (La Barbera, 2015)**
EU should be a single country.
I am in favor of a centralized management scheme of the European economy to replace the economic powers of individual states.
I see positively a more immediate action in applying European norms.
I would be in favor of the creation of one single European army to replace national armies.
I would be in favor of giving a greater political power to the EU, greater than the power of each single country.

**Attitude towards EU enlargement (La Barbera, 2015)**
I see positively the enlargement of the EU beyond the countries that have joined from the beginning.
I think that EU enlargement represents an opportunity for all of us.
EU enlargement scares me.
